# Cortical and subcortical morphological alterations in *de novo GBA*-related Parkinson’s disease

**DOI:** 10.3389/fnagi.2026.1885255

**Published:** 2026-08-12

**Authors:** Jingru Ren, Yi Xing, Hao Zhou, Xinyu Hong, Xiaofang Huang, Weiguo Liu

**Affiliations:** Department of Neurology, The Affiliated Brain Hospital of Nanjing Medical University, Nanjing, China

**Keywords:** amygdala subfield, cortical thickness, cortical volume, GBA, Parkinson’s disease

## Abstract

**Background:**

This study aimed to investigate cortical and subcortical changes in *GBA*-related Parkinson’s disease (*GBA*-PD) patients and evaluate the ability of these structural changes to identify patients with *GBA*-PD.

**Methods:**

T1-weighted magnetic resonance imaging images were obtained for 86 participants, including 19 *GBA*-PD, 42 idiopathic PD (iPD) cases, and 25 healthy controls (HCs). Cortical thickness, cortical volume, and subcortical volume (including amygdala volume) were calculated to identify cortical and subcortical morphological alterations and correlated with cognitive function. To reduce overfitting, classification was performed using least absolute shrinkage and selection operator (LASSO)-penalized logistic regression with nested stratified 5-fold cross-validation.

**Results:**

Compared with iPD patients, *GBA*-PD patients had reduced cortical thickness in the left superior temporal sulcus (STS.L) and left superior occipital gyrus (SOG.L) as well as reduced cortical volume in the SOG.L and right amygdala subregion, including the basal nucleus, accessory basal nucleus, cortico-amygdaloid transition area, and paralaminar nucleus. Cortical thickness and cortical volume in the SOG.L, right basal nucleus, and right paralaminar nucleus were negatively correlated with memory and visuospatial function, respectively. After internal cross-validation, the exploratory LASSO-based model combining age, sex, and significant cortical and subcortical morphometric features showed moderate discrimination between *GBA*-PD and iPD, with an area under the curve of 0.821, sensitivity of 56.3%, and specificity of 88.1%.

**Conclusion:**

*GBA*-PD patients showed distinct patterns of cortical thickness and volume thinning, as well as amygdala subregional atrophy. These cortical and subcortical changes may contribute to future exploratory phenotypic characterization or patient stratification, but validation in larger independent cohorts is required.

## Introduction

Parkinson’s disease (PD) is a heterogeneous neurodegenerative disorder associated with various clinical manifestations, including motor and non-motor symptoms (NMSs; Armstrong and Okun, 2020; Bloem et al., 2021). Heterozygous variants of the glucocerebrosidase gene (*GBA*; OMIM 606463), which encodes lysosomal glucocerebrosidase (GCase), are recognized as the most common and significant genetic risk factor for PD worldwide (Aharon-Peretz et al., 2004; Sidransky et al., 2009). Compared to idiopathic PD (iPD), *GBA*-related PD (*GBA*-PD) is characterized by earlier onset, more severe motor symptoms, a higher incidence of NMSs (especially cognitive impairment), more rapid motor and cognitive deterioration, and lower survival (Stoker et al., 2020; Zhao et al., 2020; Ortega et al., 2021; Ren et al., 2022, 2023). However, the neural mechanisms underlying these distinct clinical manifestations are incompletely elucidated. Additionally, given the potential relevance of *GBA* variants to disease prognosis and therapeutic applications, it is critical to identify biomarkers associated with *GBA*-PD.

Only a few studies to date have investigated structural brain changes in *GBA*-PD patients (Filippi et al., 2022). One study showed that cortical thickness did not differ significantly between patients with iPD and *GBA*-PD (Thaler et al., 2018). However, a recent study reported thinner cortical thickness in the left temporal, parietal, and occipital gyri in a small sample (*n* = 10) of *GBA*-PD patients relative to iPD patients (Leocadi et al., 2022). These inconsistencies motivated us to investigate cortical thickness changes in *GBA*-PD. Furthermore, cortical volume patterns, an important indicator that carries unique morphological information about the cerebral cortex, remain unevaluated in *GBA*-PD patients.

The amygdala is a major limbic structure involved in emotion, olfaction, memory modulation, and cognitive-affective integration, and amygdala pathology has been linked to NMSs in PD (Harding et al., 2002; Wang et al., 2023). This is particularly relevant to *GBA*-PD, which is often associated with greater cognitive and neuropsychiatric burden than iPD (Brockmann et al., 2011; Swan et al., 2016). With advances in automated magnetic resonance imaging (MRI) segmentation, amygdala nuclei can now be quantified *in vivo*, enabling more detailed assessment beyond whole-amygdala volume (Saygin et al., 2017). Recent studies have suggested that amygdala subregional alterations are associated with cognitive impairment and pre-decline cognitive vulnerability in PD, including changes involving the cortico-amygdaloid transition area (Ay et al., 2023; Seo et al., 2025). However, amygdala subfield morphometry remains insufficiently characterized in *de novo GBA*-PD.

Investigating cortical and subcortical changes in *GBA*-PD patients may thus help elucidate why *GBA*-PD patients exhibit different clinical features than iPD. To the best of our knowledge, there are no reports on cortical and subcortical changes that differentiate patients with *GBA*-PD from those with iPD. To address these research gaps, the present study explored cortical alterations, including cortical thickness and cortical volume, as well as subcortical structural volume alterations — particularly the amygdala subfield — in patients with newly diagnosed *GBA*-PD. We also examined the ability of significant alterations in cortical and subcortical metrics to distinguish *GBA*-PD from iPD.

## Materials and methods

### Participants

This study was conducted at the Department of Neurology, Affiliated Brain Hospital of Nanjing Medical University. The inclusion criteria for PD patients were: (1) newly diagnosed with PD based on the United Kingdom Parkinson’s Disease Society Brain Bank clinical diagnostic criteria (Gibb and Lees, 1988); (2) drug naïve; (3) entire *GBA* gene testing to avoid interference from the adjacent pseudogene *GBAP1*; and (4) at least 1 year of follow-up. The exclusion criteria were: (1) the presence of atypical or secondary parkinsonism; (2) fulfillment of the Movement Disorder Society (MDS) PD dementia (PDD) clinical diagnostic criteria (Emre et al., 2007); (3) MRI scans of the brain showing clinically significant lesions, including cerebral vascular disease or intracranial masses; and (4) a history of serious chronic medical conditions, such as heart or renal failure, or complications of diabetes.

The *GBA*-PD group included patients with PD harboring any of the detected *GBA* variants; additional details of the 19 *GBA*-PD patients are provided in Supplementary Table 1. For the analysis, 42 iPD cases were matched with 19 *GBA*-PD cases on age, age of onset, sex, years of education, Unified Parkinson’s Disease Rating Scale (UPDRS) Part III score, and modified Hoehn and Yahr (H-Y) stage. In addition, 25 sex- and age-matched healthy controls (HCs) without neurologic, psychiatric, or other disorders were recruited from the community through advertisements.

This study was approved by the Medical Ethics Committee of the Affiliated Brain Hospital of Nanjing Medical University. All participants provided written informed consent before the start of the study.

### Clinical evaluation

Motor impairment and severity were assessed using the UPDRS Part II and III and modified Hoehn-Yahr (H-Y) stages. Mood, sleep state, and NMSs were assessed using the Hamilton Depression Scale (HAMD) and Hamilton Anxiety Scale (HAMA), Parkinson’s Disease Sleep Scale (PDSS), and Non-Motor Symptoms Questionnaire (NMSQuest), respectively. Overall cognitive function was evaluated using the Mini-Mental State Examination (MMSE) and Montreal Cognitive Assessment (MoCA). For participants with ≤12 years of education, the total MoCA score (if < 30) was increased by 1 point (Nasreddine et al., 2005).

Five cognitive domains were assessed using ≥2 tests, as follows: (1) attention/working memory: Digit Span Test (DST), Trail Making Test A (TMT-A), and Stroop Color-Word Test (SCWT); (2) executive function: Trail Making Test B (TMT-B), Clock Drawing Test (CDT), and Animal Fluency Test (AFT); (3) language: Wechsler Adult Intelligence Scale III (WAIS-III) Similarities Test and Boston Naming Test (BNT); (4) memory: Auditory Verbal Learning Test (AVLT) and Logical Memory Test (LMT); and (5) visuospatial function: Benton’s Judgment of Line Orientation Test (JLOT) and Hooper Visual Organization Test (HVOT). If desired, test scores can be inverted so that higher scores indicate better cognitive performance. To assess performance in each cognitive domain, the z-scores for tests belonging to the same cognitive domain were summed and then converted to z-scores to create domain z-scores.

### MRI data acquisition

T1-weighted (T1w) images were acquired using a sagittal three-dimensional spoiled gradient echo (SPGR) sequence on the same 3.0 Tesla Verio Siemens scanner at the Affiliated Brain Hospital of Nanjing Medical University. The scanner parameters were as follows: repetition time (TR) = 2530 ms; echo time (TE) = 3.34 ms; flip angle = 7 degrees; number of slices = 128; slice thickness = 1.33 mm; gap = 0.5 mm; matrix = 256 × 192; field of view = 256 mm × 256 mm; and bandwidth = 180 HZ/PX.

### MRI data preprocessing

Data preprocessing was performed using the toolbox for Data Processing & Analysis for Brain Imaging on Surface (DPABISurf 2.0) (Yan et al., 2021), which is based on fMRIPrep (Esteban et al., 2019), FreeSurfer (Dale et al., 1999), ANTs (Avants et al., 2008), FSL (Jenkinson et al., 2002), AFNI (Cox, 1996), SPM (Ashburner, 2012), PALM (Winkler et al., 2016), GNU Parallel, MATLAB R2022a (the MathWorks, Inc., Natick, Massachusetts, United States), Docker^1^, and DPABI 7.0 (Yan et al., 2016).

The data preprocessing pipeline used in this study comprises the following steps: (1) Conversion of the data to Brain Imaging Data Structure (BIDS) format; (2) Calling of the fMRIPrep 22.1.0 docker; and (3) Preprocessing the anatomical data as follows: intensity non-uniformity (INU) correction for T1w images using N4BiasFieldCorrection; skull stripping of T1w-reference using ANTs; brain tissue segmentation of cerebrospinal fluid (CSF), white matter (WM), and gray matter (GM) using fast (FSL 6.0.5.1) on brain-extracted T1w; brain surface reconstruction using recon-all (FreeSurfer 7.2.0); and spatial normalization to the ICBM 152 Non-linear Asymmetrical template version 2009c (MNI152NLin2009cAsym) using non-linear registration.

After processing the anatomical images, various metrics including cortical thickness and cortical volume were automatically calculated and smoothed with a 10 mm full-width at a half-maximum Gaussian kernel. GM volume was calculated for bilateral subcortical regions including the thalamus, caudate, putamen, pallidum, hippocampus, amygdala, and accumbens, as well as estimated total intracranial volume (eTIV).

### Segmentation and analysis of amygdala subfields

To explore which amygdala subregions were atrophic, the bilateral amygdala was segmented using the automated amygdala segmentation algorithm in FreeSurfer 7.3, which is based on a probabilistic atlas of ultra-high-resolution MRI data and a modified version of Van Leemput’s algorithm (Saygin et al., 2017). Subsequently, GM volumes were calculated for the following nine amygdala subregions in each hemisphere: lateral nucleus, basal nucleus, accessory basal nucleus, anterior amygdaloid area, central nucleus, medial nucleus, cortical nucleus, cortico-amygdaloid transition area, and paralaminar nucleus.

### Statistical analysis

Statistical analyses were performed using SPSS version 27.0 (SPSS, Inc.) with a two-tailed significance level of *p* < 0.05. The chi-square test, one-way analysis of variance (ANOVA), or Kruskal-Wallis H test were used to compare demographic and clinical data between the three groups, followed by *post hoc* analyses using Bonferroni correction. The two-sample *t*-test or Mann-Whitney U test was used for comparisons between the iPD and *GBA*-PD groups. Analysis of covariance (ANCOVA) was used to evaluate cortical thickness and cortical volume differences among the HCs, iPD, and *GBA*-PD groups controlling for age, sex, and years of education. The significance level was set with Monte Carlo simulation correction (vertex-level *p* < 0.001, cluster-level *p* < 0.025 for each hemisphere) using the DPABISurf 2.0. ANCOVA was also used to determine significant regional cortical thickness and volume differences among the three groups with age, sex, and education included as covariates, followed by *post hoc* analysis with Bonferroni adjustment. The general linear model (GLM) was used to compare subcortical volumes — especially amygdala subregion volumes — among the three groups, adjusting for age, sex, years of education, and eTIV. In cases of multiple comparisons, false discovery rate (FDR) correction (Benjamini-Hochberg procedure) was applied. *Post hoc* tests for pairwise comparisons were performed using Bonferroni correction when the FDR-*p* value was <0.05. Partial correlation analyses were performed to examine relationships between cortical and subcortical alterations and cognitive function in newly diagnosed PD patients, with age, sex, and education as covariates. Additionally, eTIV was treated as an extra covariate for subcortical gray matter volume.

Lastly, to reduce overfitting, the classification model for receiver operating characteristic (ROC) analysis was constructed using least absolute shrinkage and selection operator (LASSO)-penalized logistic regression with nested stratified 5-fold cross-validation in Python version 3.12. Candidate predictors included imaging features showing significant differences between the *GBA*-PD and iPD groups, together with age and sex. Continuous predictors were standardized within each training fold. The LASSO penalty parameter was selected in the inner 5-fold loop, and performance was evaluated using out-of-fold predictions from the outer 5-fold loop. The classification threshold was determined by the Youden index in each training fold and applied to the corresponding held-out test fold. Model performance was summarized using cross-validated area under the curve (AUC), sensitivity, specificity, positive predictive value (PPV), and negative predictive value (NPV). The 95% confidence interval (CI) for the AUC was estimated using stratified bootstrap resampling of the out-of-fold predicted probabilities.

## Results

### Demographic and clinical characteristics

The demographic and clinical characteristics of 19 *GBA*-PD patients, 42 iPD patients, and 25 HCs are summarized in Table 1. As shown, there were no significant group differences in terms of age, sex, years of education, or MMSE scores. There were no statistically significant differences between the *GBA*-PD and iPD groups in terms of demographic and clinical data, including the battery of neuropsychological test scores, except for JLOT scores. Notably, the HAMD, HAMA, and MoCA scores differed significantly among the three groups. *Post hoc* analysis showed that GBA-PD and iPD groups exhibited more severe HAMD, HAMA, and MoCA scores compared with HCs.

**TABLE 1:** Demographic information and clinical characteristics of participants.

Variables	HCs (*n* = 25)	iPD (*n* = 42)	*GBA*-PD (*n* = 19)	*P-*value	*post hoc*
Age (years)	56.5 ± 5.2	54.5 ± 5.1	52.2 ± 8.5	0.352	
Sex (male/female)	10/15	26/16	11/8	0.208	
Formal education (years)	11.3 ± 2.5	10.4 ± 2.9	9.7 ± 3.9	0.195	
Age at onset (years)		52.3 ± 5.0	50.5 ± 8.6	0.387	
Disease duration (years)		2.2 ± 1.9	1.7 ± 1.5	0.233	
UPDRS part II		6.8 ± 3.5	8.0 ± 3.6	0.236	
UPDRS part III		20.3 ± 8.2	24.2 ± 10.9	0.252	
H-Y stage		1.6 ± 0.5	1.6 ± 0.4	0.987	
NMSQuest		7.1 ± 3.6	9.7 ± 6.7	0.257	
PDSS		129.6 ± 24.8	114.4 ± 27.8	0.081	
HAMD	2.4 ± 4.2	9.3 ± 6.1	11.6 ± 10.1	**<0.001**	**<0.001^a,b^**
HAMA	1.8 ± 3.0	6.2 ± 4.4	9.4 ± 8.8	**<0.001**	**<0.001^a,b^**
MMSE	28.8 ± 1.3	27.8 ± 2.4	27.6 ± 2.2	0.098	
MoCA	26.3 ± 1.9	23.9 ± 3.4	23.9 ± 3.6	**0.006**	**0.008^a^, 0.033^b^**
Attention/working					
DST		11.9 ± 2.1	12.4 ± 2.3	0.474	
TMT-A (second)		84.8 ± 34.1	81.4 ± 33.1	0.695	
SCWT-C-right		47.2 ± 4.7	48.3 ± 4.6	0.052	
Executive function					
TMT-B (second)		182.9 ± 73.9	153.7 ± 32.8	0.223	
CDT		8.2 ± 2.6	8.7 ± 2.3	0.441	
AFT		18.2 ± 4.7	20.1 ± 5.2	0.191	
Language					
Similarities		16.7 ± 3.8	16.7 ± 4.4	0.982	
BNT		24.1 ± 3.2	24.7 ± 3.1	0.687	
Memory					
AVLT-delayed recall		5.2 ± 2.5	5.9 ± 2.2	0.319	
LMT-delayed recall		5.3 ± 2.2	6.5 ± 2.2	0.066	
Visuospatial function					
JLOT		24.0 ± 3.0	25.5 ± 2.9	**0.046**	
HVOT		15.5 ± 4.2	15.1 ± 4.3	0.780	

Data represent the mean ± standard deviation (SD). Only 16 *GBA*-PD and 42 iPD patients completed the various neuropsychological tests. Differences and *p*-values between the HCs, iPD, and *GBA*-PD groups were computed using the chi-square test, one-way analysis of variance (ANOVA), or Kruskal-Wallis H-test, followed by *post hoc* analysis with Bonferroni correction. Differences and *p*-values between the iPD and *GBA*-PD groups were computed using the two-sample *t*-test or Mann-Whitney U-test. Statistically significant differences (*p* < 0.05) are indicated in bold.

^a^*Post hoc* analysis showed statistically significant differences between the iPD and HCs groups.

^b^*Post hoc* analysis showed statistically significant differences between the *GBA*-PD and HCs groups. AFT, Animal Fluency Test; AVLT, Auditory Verbal Learning Test; BNT, Boston Naming Test; CDT, Clock Drawing Test; DST, Digit Span Backward Test; *GBA*-PD, *GBA*-related PD; HAMA, Hamilton Anxiety Scale; HAMD, Hamilton Depression Scale; HCs, healthy controls; HVOT, Hooper Visual Organization Test; H-Y, Hoehn and Yahr; iPD, idiopathic PD; JLOT, Benton’s Judgment of Line Orientation Test; LMT, Logical Memory Test; MMSE, Mini-Mental State Examination; MoCA, Montreal Cognitive Assessment; NMSQuest, Non-Motor Symptoms Questionnaire; PD, Parkinson’s disease; PDSS, Parkinson’s Disease Sleep Scale; SCWT, Stroop Color-Word Test; TMT-A, Trail Making Test A; TMT-B, Trail Making Test B; UPDRS, Unified Parkinson’s Disease Rating Scale.

### Cortical thickness and cortical volume changes in *GBA*-PD and iPD

**FIGURE 1:**
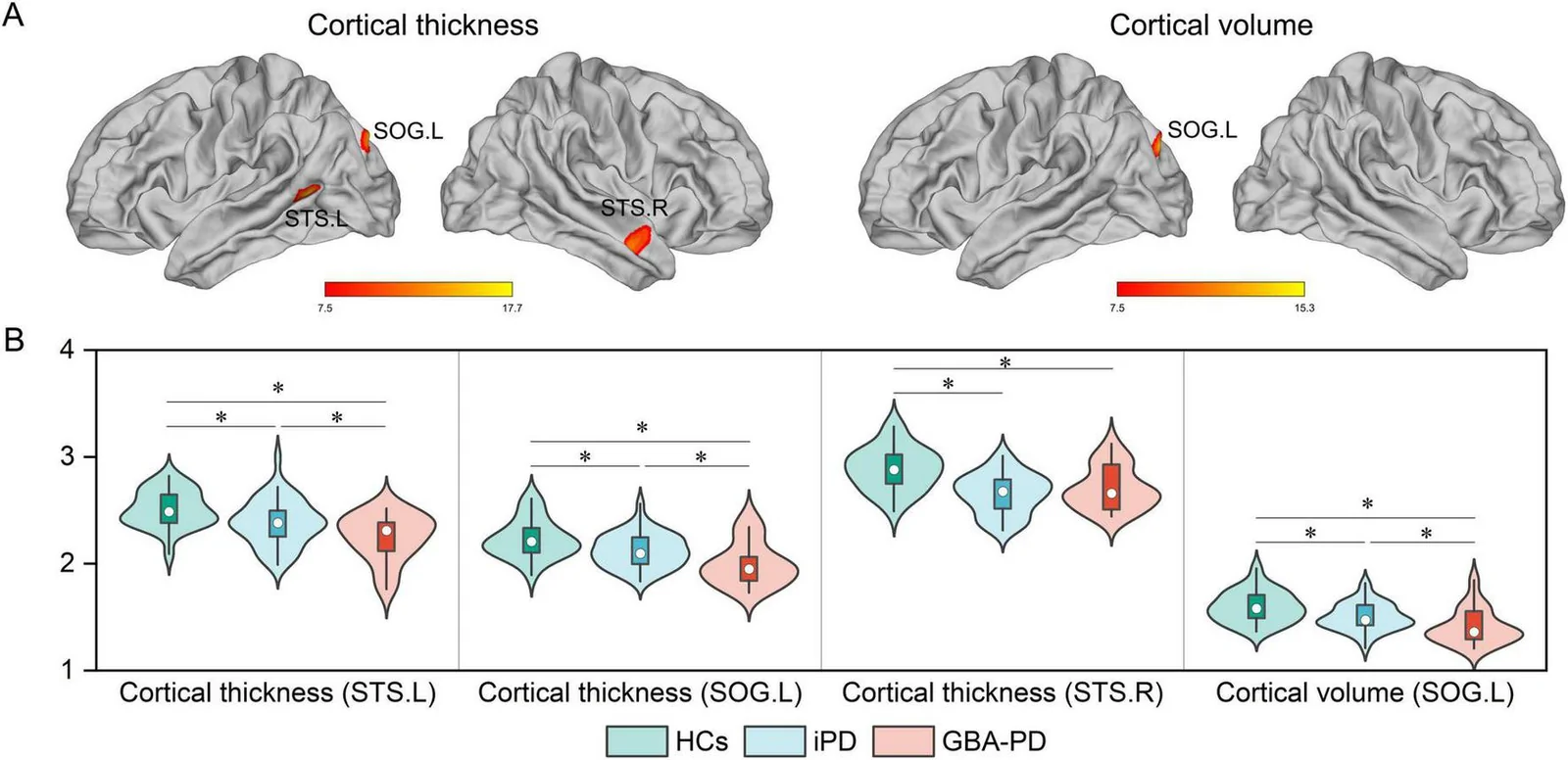
Patterns of cortical alterations among the HCs, iPD, and *GBA*-PD groups. **(A)** Brain regions showed significant differences in cortical thickness and cortical volume across the three groups. The results were corrected for multiple comparisons using Monte Carlo simulation correction (vertex-level *p* < 0.001, cluster-level *p* < 0.025 for each hemisphere) including age, sex, and years of education as covariates. **(B)** Group comparisons of regional cortical thickness and volume were calculated using ANCOVA followed by *post hoc* analysis with Bonferroni adjustment, controlling for age, sex, and education. *p < 0.05. ANCOVA, Analysis of covariance; *GBA*-PD, *GBA*-related PD; HCs, healthy controls; iPD, idiopathic PD; L, left; PD, Parkinson’s disease; SOG, superior occipital gyrus; STS, superior temporal sulcus.

Analysis of covariance analysis revealed significant cortical thickness alterations across the three groups in the left superior temporal sulcus (STS.L), left superior occipital gyrus (SOG.L), and right superior temporal sulcus (STS.R), as well as significant cortical volume alterations in the SOG.L (Figure 1A and Supplementary Table 2). Compared to HCs, *GBA*-PD and iPD patients showed decreased cortical thickness in the STS.L, SOG.L, and STS.R and decreased cortical volume in the SOG.L. Notably, *GBA*-PD patients had reduced cortical thickness in STS.L and SOG.L, and reduced cortical volume in SOG.L, compared to iPD patients (Figure 1B).

### Subcortical volumetric changes in *GBA*-PD and iPD

**FIGURE 2:**
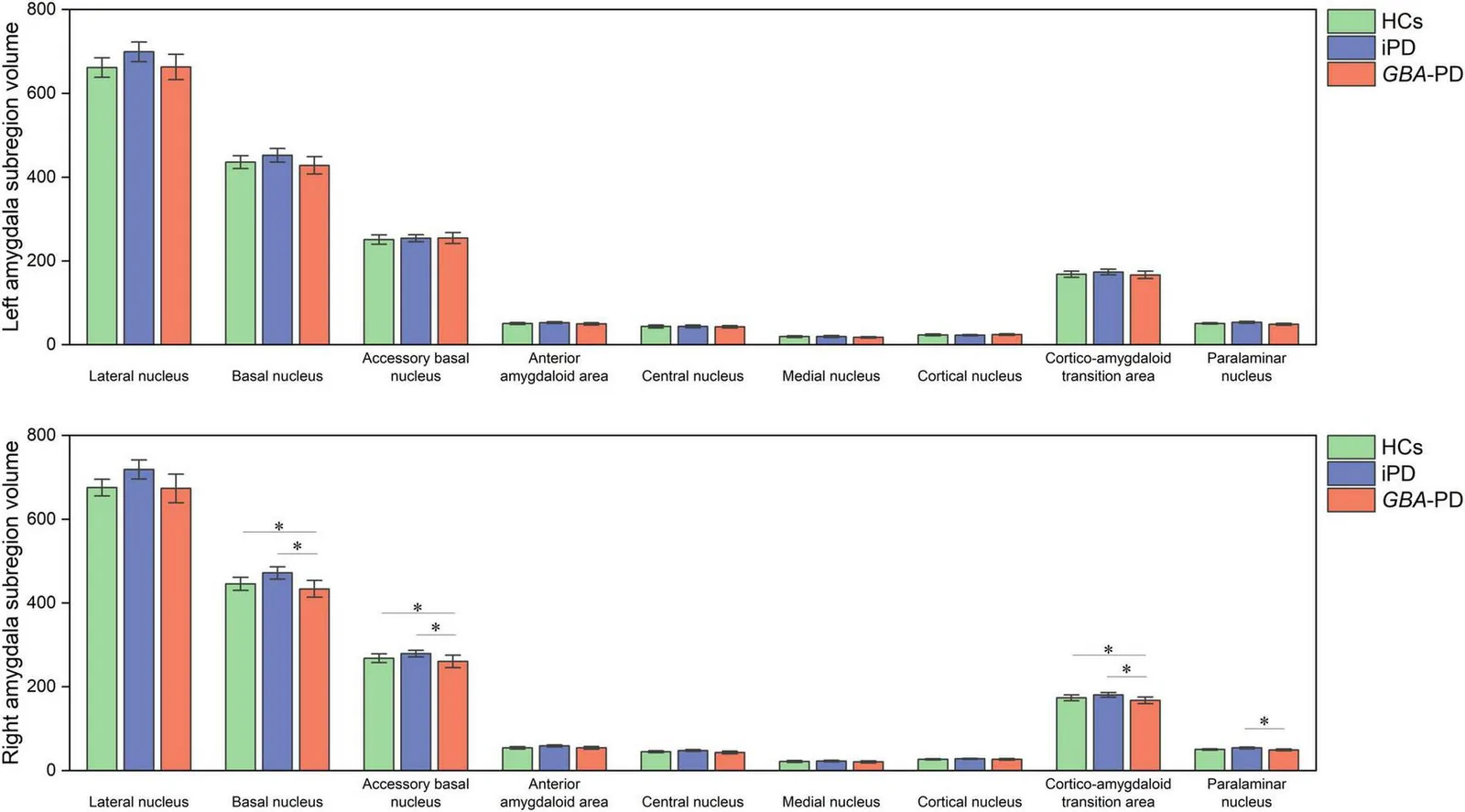
Comparison of amygdala subfield volumes across the *GBA*-PD, iPD, and HCs groups. Error bars represent 95% confidence intervals for the mean values. *P*-values were obtained using the general linear model (GLM) with age, sex, education, and estimated total intracranial volume as covariates. When the *p*-values survived false discovery rate (FDR) correction, *post hoc* tests for pairwise comparisons were performed. *p < 0.05. *GBA*-PD, *GBA*-related PD; HCs, healthy controls; iPD, idiopathic PD; PD, Parkinson’s disease.

Regarding subcortical structure volumes, only the right amygdala (*p* = 0.012) showed a significant difference between the three groups, although this result did not survive FDR correction (Supplementary Table 3). Further comparisons revealed differences in the volumes of the right basal nucleus, right accessory basal nucleus, right cortico-amygdaloid transition area, and right paralaminar nucleus between the three groups after FDR correction (Figure 2 and Supplementary Table 4). *Post hoc* tests showed that the *GBA*-PD group had reduced volumes in the right basal nucleus, right accessory basal nucleus, and right cortico-amygdaloid transition area compared with the iPD and HCs groups. The volume of the right paralaminar nucleus was also lower in *GBA*-PD patients than in iPD patients.

### Relationships between cortical and subcortical alterations and cognitive function in PD

**FIGURE 3:**
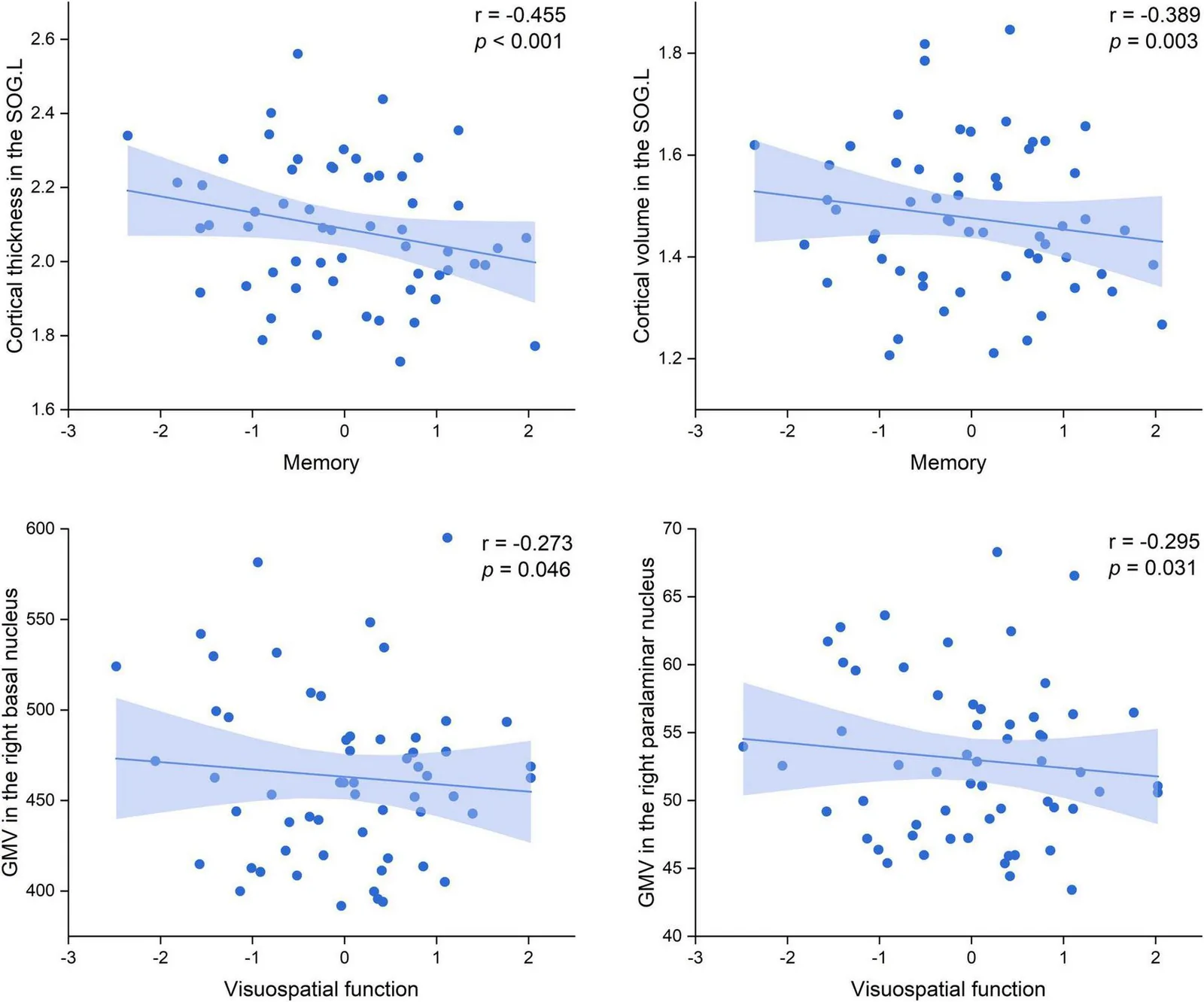
Correlation between cortical and subcortical alterations and cognitive performance in *de novo* PD patients. Partial correlation analyses included age, sex, and education. Estimated total intracranial volume was included as an extra covariate for subcortical gray matter volume. GMV, gray matter volume; L, left; PD, Parkinson’s disease; SOG, superior occipital gyrus.

Figure 3 depicts the association between cortical and subcortical morphological alterations and cognitive function in patients with newly diagnosed PD. Cortical thickness and SOG.L cortical volume were significantly negatively correlated with memory (*r* = −0.455, *p* < 0.001; *r* = −0.389, *p* = 0.003; respectively). In addition, the right basal nucleus and right paralaminar nucleus showed significant negative correlations with visuospatial function (*r* = −0.273, *p* = 0.046; *r* = −0.295, *p* = 0.031; respectively).

### Distinguishing *GBA*-PD from iPD patients using ROC analysis

**FIGURE 4:**
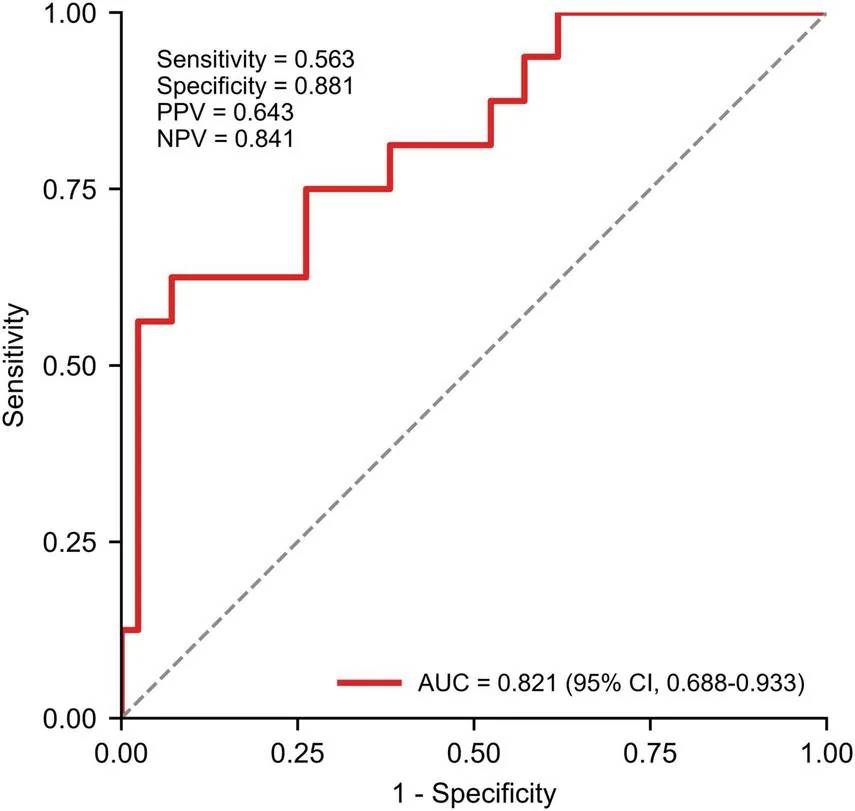
Receiver operating characteristic analysis distinguishing *GBA*-PD patients from iPD patients. AUC, area under the curve; CI, confidence interval; *GBA*-PD, *GBA*-related PD; iPD, idiopathic PD; PD, Parkinson’s disease; PPV, positive predictive value; NPV, negative predictive value.

Next, the LASSO-penalized logistic regression model included seven imaging features showing significant differences between the *GBA*-PD and iPD groups, together with age and sex. These imaging features included cortical thickness in the STS.L and SOG.L, cortical volume in the SOG.L, right basal nucleus, right accessory basal nucleus, right cortico-amygdaloid transition area, and right paralaminar nucleus. In nested stratified cross-validation, the model achieved an out-of-fold AUC of 0.821 (95% CI, 0.688–0.933), with sensitivity of 56.3%, specificity of 88.1%, PPV of 64.3%, and NPV of 84.1% (Figure 4). In the final refitted LASSO model, all candidate predictors were retained except right accessory basal nucleus, which was shrunk to zero.

## Discussion

This is the first comprehensive study of structural morphological changes in *de novo GBA*-PD patients exploring the potential value of alterations in cortical thickness, cortical volume, and subcortical volume, with a focus on amygdala subregions. We found reduced cortical thickness and cortical volume in the STS.L and SOG.L, as well as reduced volumes of the basal nucleus, accessory basal nucleus, cortico-amygdaloid transition area, and paralaminar nucleus in the right amygdala subregion in *GBA*-PD patients compared with iPD patients. Further analysis showed that these changes were significantly negatively correlated with memory and visuospatial function. Importantly, in the exploratory classification analysis, age, sex, and selected cortical and subcortical morphometric features showed moderate discrimination between *de novo GBA*-PD and iPD after internal cross-validation (AUC = 0.821), suggesting that structural MRI measures may provide complementary information for characterizing *GBA*-PD.

Previous studies examining cortical changes in *GBA*-PD patients are limited and their findings are inconsistent. Thaler et al. (2018) found no significant difference in cortical thickness between patients with iPD and *GBA*-PD. However, a recent study reported that, compared with iPD patients, *GBA*-PD patients showed a left-sided general pattern of cortical thinning mainly involving the temporal, parietal, and occipital gyri (Leocadi et al., 2022). In the current study, *GBA*-PD patients showed extensive cortical thickness and volume atrophy in the STS.L and SOG.L compared with iPD patients. Inconsistencies in cortical change findings may relate to the selection of *GBA*-PD patients and whether the PD patients received dopamine replacement therapy. Specifically, our study focused on newly diagnosed, drug-naive PD patients, which may reduce confounding by dopaminergic treatment and allow characterization of early structural involvement. In addition, our cohort consisted of Chinese patients, whereas the frequency and severity distribution of *GBA* variants may differ across ethnic populations. Such population-specific heterogeneity in *GBA* variant distribution may partly contribute to variability in clinical and neuroimaging findings across studies. Thus, our findings may reflect early and relatively focal cortical vulnerability in *GBA*-PD.

This study provides the first evidence that *GBA*-PD patients have atrophy of the right amygdala subregions compared with iPD patients. A recent study reported that amygdala subregions exhibit common and unique hypoconnectivity in PD, and that hypoconnectivity of amygdala subregions is associated with various NMSs including mood, pain, olfaction, and cognition (Wang et al., 2023). Thus, the observed atrophy of the amygdala and its subregions may contribute to the extensive NMSs in *GBA*-PD patients. Cognitive impairment is a specific clinical signature of *GBA*-PD. Ay et al. (2023) previously reported that the cortico-amygdaloid transition area volume in amygdala subregions was smaller in PD patients with cognitive impairment. However, in the present study, *GBA*-PD patients showed atrophy in the basal nucleus, accessory basal nucleus, cortico-amygdaloid transition area, and paralaminar nucleus.

In our cohort of newly diagnosed PD patients, both cortical thickness and volume of the SOG.L were significantly negatively correlated with memory performance. We also found that the volumes of the right basal and paralaminar nuclei of the amygdala were negatively associated with visuospatial function in PD patients. These inverse associations may be related to the early-stage nature of our cohort, in which regional morphometric alterations may reflect not only established atrophy but also dynamic pathological remodeling, glial/neuroinflammatory responses, and compensatory or maladaptive network reorganization (Aarsland et al., 2021; Tansey et al., 2022; Schumacher et al., 2023). The SOG.L belongs to posterior visual-association and parieto-occipital systems, and visual/visuospatial dysfunction in PD has been linked to posterior cortical network alterations and cognitive decline (Kravitz et al., 2011; Hannaway et al., 2023). Recent studies also suggest that amygdala subregional alterations are involved in PD-related cognitive impairment, including early or pre-decline stages (Ay et al., 2023; Seo et al., 2025). Thus, these negative associations may reflect early network-level vulnerability involving visual-associative and limbic-cognitive circuits rather than a simple protective effect of larger regional volume.

Although *GBA*-PD patients showed more pronounced cortical and subcortical morphometric alterations than iPD patients, there was substantial overlap in these measures between the two groups. To further examine whether these imaging features could provide complementary discriminatory information, we performed an exploratory classification analysis. Age, sex, and selected cortical and subcortical morphometric features showed moderate discrimination between *de novo GBA*-PD and iPD after internal cross-validation (AUC = 0.821). This finding suggests that structural MRI measures capture disease-relevant neuroanatomical information and may provide complementary value for characterizing the *GBA*-PD phenotype. Importantly, these imaging findings should not be interpreted as a substitute for genetic testing, which remains the definitive method for identifying *GBA* mutation carrier status. Rather, if independently validated, structural MRI measures may provide complementary information regarding disease phenotype, neural involvement, cognitive risk, and patient stratification in future disease-modifying or targeted therapeutic trials.

This study provides the first comprehensive investigation of cortical thickness, cortical volume, and subcortical volume (especially amygdala subregions) in patients with *de novo GBA*-PD to establish a panel of neuroimaging biomarkers to distinguish *GBA*-PD from iPD. However, some limitations should be noted. First, although LASSO-penalized logistic regression with nested cross-validation was used to reduce overfitting, this remains an internal validation procedure, and the candidate imaging predictors were selected from the same cohort. Therefore, validation in larger independent cohorts is required before clinical diagnostic or stratification applications can be considered. Second, *GBA* variants are heterogeneous and may be classified into mild, severe, risk, and complex variants; therefore, the variant distribution shown in Supplementary Table 1 may have influenced the imaging findings. However, given the relatively low frequency of *GBA* variants in the Chinese population (2%–11%) (Li et al., 2020; Ren et al., 2022), the number of *GBA*-PD patients in our study was limited, precluding reliable subgroup analyses according to mutation severity. Future studies with larger samples are needed to examine variant-specific cortical and subcortical alterations in *GBA*-PD. Third, due to the cross-sectional study design, we were unable to obtain trajectories of changes in these neuroimaging biomarkers over time. Therefore, future longitudinal follow-up studies will be needed to track the neuroimaging biomarker profiles of *GBA*-PD patients to examine changes in neuroimaging biomarkers during disease progression.

In conclusion, this cross-sectional study has revealed distinct patterns of cortical thickness and volume thinning, as well as amygdala subregional atrophy, in *GBA*-PD patients compared with iPD patients. Exploratory LASSO-based classification with nested cross-validation further suggested that these imaging features provide complementary information for distinguishing *GBA*-PD from iPD. These findings support the potential value of structural MRI for phenotypic characterization and future patient stratification in *GBA*-PD, although larger independent cohorts are needed to validate their reproducibility and clinical utility.

## Data Availability

The original contributions presented in this study are included in this article/Supplementary material, further inquiries can be directed to the corresponding author.

## Footnotes

1
https://docker.com
