# Supplementary material for: Cortical and subcortical morphological alterations in de novo GBA-related Parkinson’s disease
Source: Front Aging Neurosci. 2026 Aug 12;18:1885255. doi: 10.3389/fnagi.2026.1885255 (PMC13506707; doi:10.3389/fnagi.2026.1885255)
Supplement: Supplementary file 1 [file Data_Sheet_1.docx]

**Supplementary Materials**

**Supplementary Table**

**Supplementary Table 1.** *GBA* variants identified in newly diagnosed PD patients

**Supplementary Table 2.** Brain regions showing cortical alterations among the HCs, iPD, and *GBA*-PD groups

**Supplementary Table 3.** Volume of subcortical structures(mm^3^) across the three groups

**Supplementary Table 4.** Amygdala subfield volumes (mm^3^) in study participants

**Supplementary Table 1.** *GBA* variants identified in newly diagnosed PD patients

| **Cases** | **Allele name**  **(HGVS)** | **Allele name**  **(traditional)** | **Amino acid**  **change** | **Nucleotide**  **change** | **Exon** | **Class of**  **variant** | **RS (dbSNP)** |
| --- | --- | --- | --- | --- | --- | --- | --- |
| 3 | L483P | L444P | p.Leu483Pro | c.1448T>C | 11 | severe | rs421016 |
| 2 | R202Q | R163Q | p.Arg202Gln | c.605G>A | 7 | unknown | rs398123531 |
| 1 | A33V | A(-7)V | p.Ala33Val | c.98C>T | 3 | unknown | rs776856496 |
| 1 | D448H | D409H | p.Asp448His | c.1342G>C | 10 | severe | rs1064651 |
| 1 | G241R | G202R | p.Gly241Arg | c.721G>A | 7 | severe | rs409652 |
| 1 | L105Q | L66Q | p.Leu105Gln | c.314T>A | 5 | unknown | - |
| 1 | L303I | L264I | p.Leu303Ile | c.907C>A | 8 | mild | rs1296507371 |
| 1 | L422Pfs*3 | L383Pfs*3 | p.Leu422Profs*3 | c.1265_1319del | 10 | severe | rs80356768 |
| 1 | R159W | R120W | p.Arg159Trp | c.475C>T | 6 | severe | rs439898 |
| 1 | R324H | R285H | p.Arg324His | c.971G>A | 8 | severe | rs79696831 |
| 1 | S52S | S13S | p.Ser52Ser | c.156G>A | 4 | unknown | rs756264143 |
| 1 | T107A | T68A | p.Thr107Ala | c.319A>G | 5 | unknown | - |
| 1 | V414L | V375L | p.Val414Leu | c.1240G>C | 10 | mild | rs398123528 |
| 1 | V499V | V460V | p.Val499Val | c.1497G>C | 11 | complex | rs1135675+ |
|  | A495P | A456P | p.Ala495Pro | c.1483G>C |  |  | rs368060+ |
|  | L483P | L444P | p.Leu483Pro | c.1448T>C |  |  | rs421016 |
| 1 | R202Q | R163Q | p.Arg202Gln | c.605G>A | 7-11 | complex | rs398123531+ |
|  | L483P | L444P | p.Leu483Pro | c.1448T>C |  |  | rs421016 |
| 1 | S310G | S271G | p.Ser310Gly | c.928A>G | 8-11 | complex | rs1057942+ |
|  | P491P | P452P | p.Pro452Pro | c.1473C>T |  |  | rs149257166 |

Note: The allele name (HGVS) is based on the reference sequence NM_001005741 and the Human Genome Variation Society nomenclature (HGVS; http://varnomen.hgvs.org/). The allele name (traditional) is based on the traditional nomenclature associated with Gaucher disease, referring to the processed protein with the first 39 amino acids removed.

Abbreviations: *GBA*-PD, *GBA*-related PD; PD, Parkinson's disease.

**Supplementary Table 2.** Brain regions showing cortical alterations among the HCs, iPD, and *GBA*-PD groups

| Brain regions | Abbreviations | Peak MNI coordinate | | | Peak *F* value | Cluster size (Vertices) |
| --- | --- | --- | --- | --- | --- | --- |
|  |  | x | y | z |  |  |
| Cortical thickness |  |  |  |  |  |  |
| Left superior temporal sulcus | STS.L | -52.0 | -56.2 | 5.6 | 14.9 | 395 |
| Left superior occipital gyrus | SOG.L | -17.7 | -86.3 | 31.0 | 17.7 | 329 |
| Right superior temporal sulcus | STS.R | 48.8 | -5.9 | -21.0 | 12.0 | 357 |
| Cortical volume |  |  |  |  |  |  |
| Left superior occipital gyrus | SOG.L | -20.1 | -86.3 | 28.7 | 15.3 | 395 |

*Note:* Analysis of covariance (ANCOVA) was performed with Monte Carlo simulation correction (vertex-level *p* < 0.001, cluster-level *p* < 0.025 for each hemisphere) using age, sex, and years of education as covariates.

Abbreviations: *GBA*-PD, *GBA*-related PD; HCs, healthy controls; iPD, idiopathic PD; PD, Parkinson's Disease.

**Supplementary Table 3.** Volume of subcortical structures(mm^3^) across the three groups

| Subfields | HCs | iPD | *GBA*-PD | *p* value | FDR-*p* |
| --- | --- | --- | --- | --- | --- |
| Left |  |  |  |  |  |
| Thalamus | 6667.1 ± 559.4 | 7016.5 ± 704.5 | 6872.8 ± 737.2 | 0.861 | 0.925 |
| Caudate | 3286.1 ± 368.9 | 3340.9 ± 432.2 | 3126.1 ± 399.2 | 0.071 | 0.497 |
| Putamen | 4713.5 ± 365.8 | 4915.2 ± 523.8 | 4774.5 ± 636.8 | 0.577 | 0.808 |
| Pallidum | 2027.3 ± 222.4 | 2131.6 ± 283.9 | 2100.2 ± 149.6 | 0.925 | 0.925 |
| Hippocampus | 4073.0 ± 378.8 | 4074.8 ± 406.7 | 4067.4 ± 449.5 | 0.233 | 0.652 |
| Amygdala | 1640.4 ± 169.9 | 1686.7 ± 280.0 | 1633.0 ± 227.8 | 0.353 | 0.796 |
| Accumbens | 481.2 ± 112.4 | 489.6 ± 117.8 | 497.6 ± 105.0 | 0.576 | 0.808 |
| Right |  |  |  |  |  |
| Thalamus | 6705.9 ± 591.5 | 7101 ± 865.6 | 6960 ± 793.2 | 0.914 | 0.925 |
| Caudate | 3363.0 ± 349.3 | 3424.6 ± 454.3 | 3186.7 ± 412.4 | 0.187 | 0.652 |
| Putamen | 4928.2 ± 362.8 | 5042.1 ± 493.2 | 4880.2 ± 620.3 | 0.181 | 0.652 |
| Pallidum | 1952.6 ± 245.1 | 2023.1 ± 236.3 | 1998.2 ± 158 | 0.923 | 0.925 |
| Hippocampus | 4268.1 ± 354.4 | 4361.1 ± 372.8 | 4297.9 ± 412.3 | 0.506 | 0.808 |
| Amygdala | 1768.1 ± 184.9 | 1843.5 ± 242.4 | 1692.4 ± 247.6 | **0.012** | 0.168 |
| Accumbens | 540.2 ± 94.6 | 536.5 ± 111.2 | 541.2 ± 114.5 | 0.398 | 0.796 |

*Note*: Data represent the mean ± standard deviation (SD).

*P* values were calculated using the general linear model (GLM), controlling for age, sex, years of education, and estimated total intracranial volume, followed by false discovery rate (FDR) correction (Benjamini-Hochberg procedure) for multiple testing.

The statistically significant values (*p* < 0.05) are marked in bold.

Abbreviations: *GBA*-PD, *GBA*-related PD; HCs, healthy controls; iPD, idiopathic PD; PD, Parkinson's Disease.

**Supplementary Table 4.** Amygdala subfield volumes (mm^3^) in study participants

| Subfields | HCs | iPD | *GBA*-PD | *p* value | FDR-*p* | post hoc |
| --- | --- | --- | --- | --- | --- | --- |
| Left |  |  |  |  |  |  |
| Lateral nucleus | 661.7 ± 56.0 | 699.2 ± 74.7 | 662.9 ± 62.5 | 0.184 | 0.332 |  |
| Basal nucleus | 436.1 ± 37.1 | 452.2 ± 51.3 | 428.2 ± 43.0 | 0.109 | 0.218 |  |
| Accessory basal nucleus | 250.9 ± 27.0 | 254.2 ± 26.6 | 254.8 ± 27.3 | 0.500 | 0.603 |  |
| Anterior amygdaloid area | 50.6 ± 5.9 | 52.7 ± 7.1 | 49.7 ± 5.7 | 0.278 | 0.400 |  |
| Central nucleus | 43.7 ± 8.6 | 43.7 ± 10.1 | 43.0 ± 5.8 | 0.723 | 0.746 |  |
| Medial nucleus | 19.5 ± 4.9 | 19.5 ± 7.0 | 17.6 ± 3.7 | 0.535 | 0.603 |  |
| Cortical nucleus | 23.4 ± 4.6 | 22.6 ± 4.6 | 24.2 ± 3.9 | 0.280 | 0.400 |  |
| Cortico-amygdaloid transition area | 168.3 ± 18.4 | 173.6 ± 21.8 | 166.7 ± 18.4 | 0.356 | 0.475 |  |
| Paralaminar nucleus | 50.8 ± 4.3 | 53.6 ± 7.2 | 48.9 ± 4.9 | **0.019** | 0.063 |  |
| Right |  |  |  |  |  |  |
| Lateral nucleus | 675.4 ± 48.4 | 718.7 ± 73.4 | 673.5 ± 71.0 | 0.063 | 0.149 |  |
| Basal nucleus | 445.6 ± 37.7 | 471.9 ± 46.7 | 433.7 ± 41.4 | **0.002** | **0.030** | **0.026^a^, 0.001^b^** |
| Accessory basal nucleus | 268.2 ± 25.2 | 279.2 ± 25.0 | 260.6 ± 31.2 | **0.008** | **0.032** | **0.015^a^, 0.014^b^** |
| Anterior amygdaloid area | 54.3 ± 6.2 | 58.8 ± 7.2 | 54.3 ± 6.8 | 0.067 | 0.149 |  |
| Central nucleus | 44.8 ± 5.5 | 48.0 ± 6.3 | 43.2 ± 6.7 | **0.045** | 0.129 |  |
| Medial nucleus | 21.7 ± 5.7 | 22.7 ± 6.3 | 20.6 ± 5.0 | 0.746 | 0.746 |  |
| Cortical nucleus | 26.9 ± 2.9 | 28.2 ± 3.3 | 26.8 ± 3.8 | 0.543 | 0.603 |  |
| Cortico-amygdaloid transition area | 173.7 ± 16.6 | 180.6 ± 18.4 | 167.5 ± 16.4 | **0.006** | **0.030** | **0.015^a^, 0.009^b^** |
| Paralaminar nucleus | 50.4 ± 4.2 | 54.1 ± 6.1 | 49.4 ± 4.6 | **0.005** | **0.030** | **0.004^b^** |

*Note:* Data represent the mean ± standard deviation (SD).

*P* values were calculated using the general linear model (GLM), controlling for age, sex, years of education, and estimated total intracranial volume, followed by false discovery rate (FDR) correction (Benjamini-Hochberg procedure) for multiple testing. For FDR-corrected *p* values < 0.05, post hoc tests were performed using Bonferroni adjustment for pairwise comparisons.

^a^ Post hoc analysis showed statistically significant differences between the HCs and *GBA*-PD groups.

^b^ Post hoc analysis showed statistically significant differences between the iPD and *GBA*-PD groups.

The statistically significant values (*p* < 0.05) are marked in bold.

Abbreviations: *GBA*-PD, *GBA*-related PD; HCs, healthy controls; iPD, idiopathic PD; PD, Parkinson's Disease.
